# A dataset for multimodal music information retrieval of Sotho-Tswana musical videos

**DOI:** 10.1016/j.dib.2024.110672

**Published:** 2024-06-26

**Authors:** Osondu Oguike, Mpho Primus

**Affiliations:** Institute for Intelligent Systems, University of Johannesburg, JBS Park, 69 Kingsway Avenue, Auckland Park, Johannesburg, South Africa

**Keywords:** Multimodal, Music information retrieval, Visual modality, Audio modality, Sotho-Tswana, Dataset

## Abstract

The existence of diverse traditional machine learning and deep learning models designed for various multimodal music information retrieval (MIR) applications, such as multimodal music sentiment analysis, genre classification, recommender systems, and emotion recognition, renders the machine learning and deep learning models indispensable for the MIR tasks. However, solving these tasks in a data-driven manner depends on the availability of high-quality benchmark datasets. Hence, the necessity for datasets tailored for multimodal music information retrieval applications is paramount. While a handful of multimodal datasets exist for distinct music information retrieval applications, they are not available in low-resourced languages, like Sotho-Tswana languages. In response to this gap, we introduce a novel multimodal music information retrieval dataset for various music information retrieval applications. This dataset centres on Sotho-Tswana musical videos, encompassing both textual, visual, and audio modalities specific to Sotho-Tswana musical content. The musical videos were downloaded from YouTube, but Python programs were written to process the musical videos and extract relevant spectral-based acoustic features, using different Python libraries. Annotation of the dataset was done manually by native speakers of Sotho-Tswana languages, who understand the culture and traditions of the Sotho-Tswana people. It is distinctive as, to our knowledge, no such dataset has been established until now.

Specifications Table

**Table:** 

Subject	Computer Science
Specific subject area	Multimedia The dataset consists of both textual, audio, and visual modalities of musical videos.
Type of data	Image: Sequence of images that make up the video. Video: Videos that were downloaded and segmented into fifteen-second video clips and separated into textual, audio, and visual modalities. Table: Tables in the form of CSV files, which contain different metadata, like Language, Sentiment, Genre, Lyrics, Meaning of Song, and spectral-based acoustic features (Onset Strength, Chroma STFT, Harmonic Percussive Source Separation (HPSS), Zero Crossing Rate, Mel-Frequency Cepstral Coefficients (MFCC), Spectral Centroid)
Data collection	The links to the videos were utilized for downloading from YouTube using the video downloader site, savefrom.net. Alternatively, a Python program, download.ipynb, was written to download the videos. Python programs were scripted to split the downloaded videos into fifteen-second video and audio clips. Relevant spectral-based acoustic features and images/frames from the videos were extracted using various Python packages such as moviepy, librosa, and cv2. Annotation of the dataset was performed manually by annotators who listened to and watched the downloaded videos.
Data source location	Social Media (YouTube) Institution: Institute for Intelligent Systems, University of Johannesburg City/Town/Region: Johannesburg Country: South Africa Primary data sources: YouTube
Data accessibility	Repository name: Mendeley Data Data identification number: 10.17632/7jmgfk4fd9.1 Direct URL to data: https://data.mendeley.com/datasets/7jmgfk4fd9/1 Instructions for accessing these data: The raw musical videos were downloaded from YouTube, and the textual and audio modalities were separated from the visual modality. Spectral-based acoustic features were then extracted from the audio modality. Subsequently, the downloaded videos were split into fifteen-second video segments. Afterward, these segments of video clips were further split into various images/frames, which will be used for training. Annotation of the dataset was done manually based on the following metadata: Language, Sentiment, Genre, Lyrics, and Meaning of Song. To access and utilize the dataset, follow these steps: 1. Download the dataset. 2. Utilize the URLs in the CSV files, VideoSegment.csv and AudioSegments.csv, to download the raw video clips from YouTube using the savefrom.net video downloader site. Alternatively, use the python script called download.ipynb, which has been included as part of the dataset, to download the YouTube videos. Ensure that you specify a CSV file with URL metadata in download.ipynb. The CSV file can be either VideoSegment.csv or AudioSegments.csv, which you have downloaded. 3. As the downloaded video clips have varying durations, employ the Jupyter notebook, SplitVideo.ipynb, along with the text file, split1.txt, to divide each downloaded video clip into equal fifteen-second segments of video clips. This will help to improve the performance during model training and evaluation, (Kiranyaz et al. 2006) [1]. 4. Ensure that the name of each segment of the video clip matches the corresponding name in the CSV file, VideoSegment.csv.
	5. Create a new folder named Video_Clips and store all the segmented video clips in this folder. Organize the segments of video clips so that those from the same downloaded video clip are placed in the same sub-folder. 6. Utilize the Jupyter notebook, separate.ipynb, to separate the audio modality of each segmented video clip from the video. 7. Ensure that the name of each segment of the audio file corresponds to the corresponding name in the CSV file, AudioSegments.csv. 8. Create a new folder named Audio_Clips and store all the segments of audio files in this folder. 9. Use the Jupyter notebook, Video_Images.ipynb, to generate frames/images from the segmented video clips. This will generate the CSV file called Video_Images.csv. Due to limitations in computing resources, when training the deep learning models, you may not use all the segmented video clips and segmented audio clips. If you decide to use only some of them, delete the ones that are not being used from the CSV files, VideoSegment.csv and AudioSegments.csv. However, if you are using a high-performance computer system, you can utilize all the segmented video clips and audio clips. 10. Store all the generated frames/images in a new folder named Video_Frames. 11. Depending on the MIR task you intend to perform, employ appropriate deep learning models to train the audio, textual, and visual modalities of the dataset, using the late fusion method.

## 1 Value of the Data

• To address complex music information retrieval tasks for data scientists and researchers, such as sentiment analysis, genre classification, emotion/mood recognition, and recommendation systems, diverse datasets are essential. Under-resourced languages, like Sotho-Tswana, lack these datasets, impeding progress in language processing for these linguistic communities. This dataset, emphasizing under-represented African languages, empowers researchers by offering diverse multimodal data. It levels the playing field for all languages in the realm of music information retrieval.
• The dataset acts as a technological resource for under-resourced languages, like Sotho-Tswana languages, thereby aiding in the development of technology for under-resourced languages, such as Sotho-Tswana. The methodology employed in this paper can also be extended to other under-resourced languages. The dataset can be reused to train different deep learning models for the textual, visual, and audio modalities while performing various music information retrieval tasks, such as sentiment analysis and genre classification, using the late fusion method.
• Those working on creating and enhancing models for music-related tasks can use this dataset to facilitate the development of more accurate and culturally inclusive algorithms.
• The dataset serves as a technological resource that can encourage people to continue learning Sotho-Tswana languages, thereby aiding in the preservation of endangered dialects within the under-resourced Sotho-Tswana language group.

## 2 Background

Modern multimodal music information retrieval often relies on machine learning and deep learning models, which require diverse multimodal datasets to accomplish tasks such as multimodal music sentiment analysis, multimodal music genre classification, multimodal music recommender systems, and multimodal music emotion/mood recognition. This motivates us to source and compile such a dataset so that music information retrieval tasks can be performed with Sotho-Tswana musical videos. Furthermore, in music information retrieval, a significant amount of information about the music is hidden in textual, audio, and visual modalities rather than in one modality alone.

## 3 Data Description

**Fig. 1:**
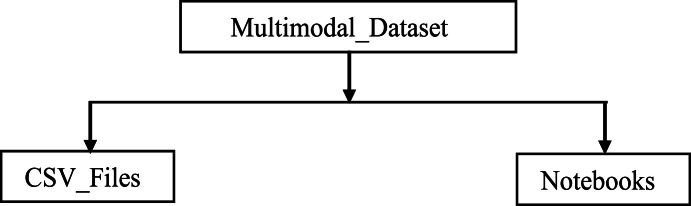
Organization of folders that store the multimodal musical dataset.

Two main folders were used to organize all the dataset files, as shown in Fig. 1. Three additional folders will be created by the user when accessing the dataset, as stated in the data accessibility section of the specifications table.

• Fig. 1: This figure illustrates the organization of the dataset into different main folders. The description of each folder follows below.
• CSV_Files:

This folder contains the following CSV files of the dataset:

1. VideoSegment.csv:

This CSV file contains the list of all 1861 segments of video clips. The metadata of this CSV file includes SN, Filename, URL, Title of Song, Type, Language, Sentiment, Lyrics, Genre, and Meaning of Song, with the following descriptions: SN is the serial number of the segments of the video clip, Filename is the name of the file used to store the video clip, URL is the Uniform Resource Locator link for the video file, Title of Song is the title of the Sotho-Tswana song as shown on YouTube, Type is the type of file (MP4), Language is the specific Sotho-Tswana language used in the song, Sentiment is the sentiment polarity of the video based on the aspects of moral and cultural values of Sotho-Tswana speaking communities (Negative, Neutral, Positive), Lyrics is the English text translated from the song, Genre is the group that the song belongs to, while Meaning of Song is a brief explanation of the meaning of the lyrics of the song. The various metadata of this CSV file were also used in the CSV file, AudioSegments.csv. The annotations of these metadata have been described in the data annotation section of 3.3.

2. AudioSegments.csv:

This CSV file contains the list of all 1861 segments of audio clips. The various metadata of this file are the same as the metadata of the CSV file, VideoSegment.csv.

3. Final_Acoustic_Features.csv:

This CSV file contains the acoustic features of all 1861 segments of audio clips. The metadata of this file are SN, Filename, Title of Song, URL, Language, Lyrics, Genre, Sentiment, Onset Strength, Chroma STFT, Harmonic Percussive Source Separation (HPSS), Zero Crossing Rate, and Mel-Frequency Cepstral Coefficients (MFCC).

4. VideoImages.csv:

This CSV file is generated when the user executes the Jupyter notebook, named ``Video_Images.ipynb.'' It contains the list of the generated images/frames corresponding to the different segments of video clips selected by the user for training. The CSV file will be used to train the deep learning model for the visual modality. Because the size of the generated images/frames of all the segmented videos is too large, we have not uploaded this CSV file. We expect the user to select the segments of video clips to use for the training. Afterward, execute the Jupyter notebook, ``Video_Images.ipynb,'' which will generate the frames/images and VideoImages.csv.

• Notebooks:

This folder contains all the Jupyter Notebook files that were used to automate some of the processes that created the dataset. It includes the following Jupyter notebook files:

1. Download.ipynb:

This Jupyter notebook contains the Python script/program that will be used to download the videos from YouTube.

2. SplitVideo.ipynb:

This Jupyter notebook contains the Python program that splits each of the downloaded 98 video clips into equal fifteen-second segments of video clips. The notebook utilizes a text file called split1.txt, which specifies the various time durations for splitting a downloaded video depending on its size. The reason for splitting the videos is to improve the performance during model training and evaluation, (Kiranyaz et al. 2006) [1].

3. Separate.ipynb:

This Jupyter notebook contains the Python program that separates the audio modality from each of the segmented video clips.

4. Features.ipynb:

This Jupyter notebook contains the Python program used to extract the different spectral-based acoustic features of all the segmented audio clips.

5. Video_Images.ipynb:

This Jupyter notebook contains the Python program used to generate the frames/images of the segments of video clips. It also generates the CSV file called VideoImages.csv, which is used to train the deep learning model for the visual modality.

## 4 Experimental Design, Materials and Methods

### 4.1 Experimental design

**Fig. 2:**
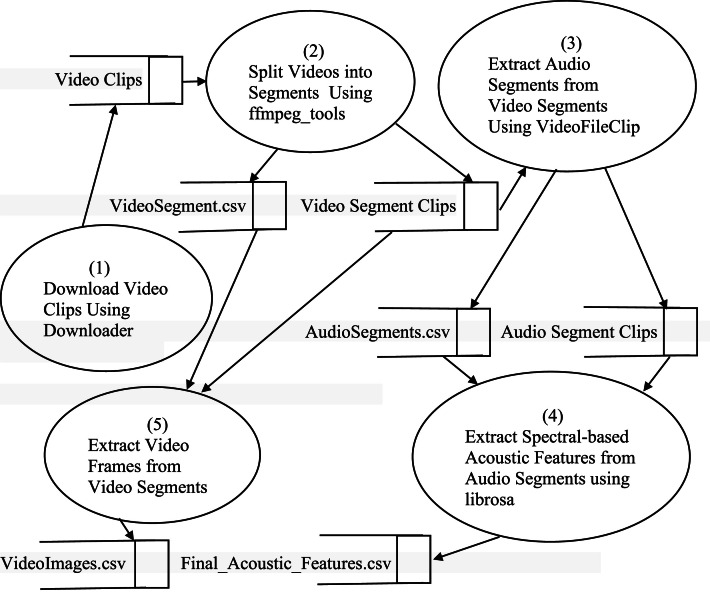
Data flow diagram of the processes that created the dataset.

The experiment used to generate the dataset consists of writing and executing Python programs on a Jupyter notebook. The description of the experiment that generated the dataset has been illustrated in the Data Flow Diagram in Fig. 2.

The dataset creation process was initiated in Process (1) by downloading raw video clips using the video downloader site, savefrom.net, and search phrases, or using the Python script, download.ipynb, to download the videos from YouTube. The Python package Pytube was used to write the Python script, download.ipynb. In Process (2), the downloaded raw video clips, numbering 98, were segmented into various video segments, each lasting fifteen seconds. This segmentation was achieved using the Python package ffmpeg_tools within the moviepy library. As depicted in the data flow diagram in Fig. 2, this process produced a CSV file named “VideoSegment.csv” and different segmented video clips.

In Process (3), the video segment clips were utilized to extract corresponding audio segment clips. This process also generated a list of all the produced audio segment clips, which was saved as a CSV file named “AudioSegments.csv.” The extraction was carried out using VideoFileClip and write.audiofile, both components of the moviepy library.

In Process (4), the CSV file generated in the previous process and the segmented audio clips were employed to extract various spectral-based acoustic features for each segmented audio clip. This extraction was performed using librosa, another Python package. The resulting spectral-based acoustic features were stored in a CSV file named “Final_Acoustic_Features.csv.”

In Process (5), the Python package CV2 was employed to extract video frames/images from the segmented video clips. Simultaneously, a list of all generated video frames/images was compiled and stored in a CSV file named “VideoImage.csv.” This CSV file, “VideoImage.csv,” will be utilized to train appropriate deep learning models for the textual and visual modalities. Conversely, the CSV file “Final_Acoustic_Features.csv” will be employed to train a suitable deep learning model for the audio modality.

However, depending on the Music Information Retrieval (MIR) application, the user may choose to extract relevant spectral-based acoustic features directly from the segmented audio clips instead of using “Final_Acoustic_Features.csv.” In that case, an appropriate notebook, which has been included, will be utilized to separate the audio segments from the video segments. Subsequently, acoustic features can be extracted during model training. The arrows in the data flow diagram of Fig. 2 show the flow of data into the various processes.

## 5 Materials

While most music information retrieval datasets have focused on Western music, there exists a database of African music, such as the archive of the Royal Museum of Central Africa in Tervuren, Belgium, which holds one of the world's largest collections of audio music from Central Africa (Moelants et al. [2]; Matthé et al. [3]; Antonopoulos et al. [4]). Another non-Western music corpus (dataset) for music information retrieval is the one collected in the CompMusic project, which consists of five different music cultures: Arab-Andalusian (Maghreb), Beijing Opera (China), Turkish Makam (Turkey), Hindustani (North-India), and Carnatic (South-India), (Serra 2014) [5]. The dataset is an audio recording with appropriate information that covers varieties of melodies and rhythms present in each musical culture.

While Moelants et al. [2] used a sample of the African music database for pitch and scale analysis, Matthé et al. [3] used the same African music database for flexible querying based on the needs of the user, and Antonopoulos et al. [4] used a sample of the same African music database for music retrieval based on rhythmic similarity with a sample of Greek traditional dance music. On the other hand, the audio dataset of the CompMusic project was aligned so that musically meaningful features related to melody and rhythm would be extracted from the audio dataset, (Serra [5]).

To our knowledge, based on the evidenced literature, there does not exist a multimodal dataset of diverse Sotho-Tswana musical videos that can be used for different multimodal MIR tasks, like multimodal music sentiment analysis, multimodal music genre classification, and multimodal music emotion recognition of Sotho-Tswana musical videos. Our dataset concentrates on textual (lyrics), audio (voice), and visual (pictures) modalities, recognizing the inherent multimodal information present in videos through lyrics, audio, and visual channels.

In a manner akin to our dataset, diverse multimodal datasets have directed their emphasis towards distinct modalities. Pandeya et al. [6] delved into the audio and visual modalities within musical videos, while Weiß et al. [7] focused their attention on lyrics (text), sheet music (visual images), and symbolic data modalities. This diversity in modality focus across various datasets contributes to a richer landscape for multimodal research, accommodating the multifaceted nature of information present in different types of multimedia content.

In their investigation, Nishikawa et al. [8] utilized two modalities, namely lyric and audio modalities, to estimate musical mood. Similarly, Zalkow et al. [9] examined symbolic encoding and audio modalities. The selection of modalities is contingent upon the authors, yet the overarching concept centres around the utilization of diverse modalities for music information retrieval (MIR).

Much like our dataset, specifically tailored for multimodal music information retrieval (MIR), several other multimodal music datasets have been formulated for various applications. For instance, Weiß et al. [7] curated a dataset comprising 24 songs from Franz Schubert's Winterreise, composed in 1827, which holds significance in the domains of music processing, music theory, and historical musicology.

Additional noteworthy datasets include the Musical Theme Dataset (MTD), introduced by Zalkow et al. [9], catering to MIR research needs. The CMU Multimodal Opinion Sentiment and Emotion Intensity (CMU-MOSEI) dataset, scrutinized for the analysis of human multimodal language by Zadeh et al. [10], and the Multimodal Opinion level Sentiment Intensity dataset (MOSI), applied in the analysis of online opinion videos as presented by Zadeh et al. [11], contribute substantially to the advancement of research in multimodal music information retrieval across diverse applications.

The Multimodal Sentiment Analysis Challenge, 2022, conducted in Lisbon, Portugal on October 10, 2022, emphasized the generation of three multimodal datasets for the detection of humour, emotional reactions, and stress. Despite these datasets not being centred around musical video content, as elucidated by Lukas Christ et al. [12], the CMU-MOSEAS (CMU Multimodal Opinion Sentiment, Emotions, and Attributes) dataset was crafted specifically for sentiment and emotion analysis, as outlined by Zadeh et al. [13].

In concordance with our dataset, numerous multimodal datasets, such as MOSI and CMU-MOSEI, derived from YouTube, have been employed to facilitate segmentation and subjectivity at the opinion level (Zadeh et al. [11]; Zadeh et al. [10]). In contrast to our dataset, the CH-SIMS dataset was meticulously curated to encompass both unimodal and multimodal aspects. This distinctive feature necessitated the independent annotation of unimodal and multimodal components (Yu et al. [14]).

The construction of the audio modality component within our dataset involved the utilization of acoustic features, like Mel Frequency Cepstral Coefficients (MFCC), recognized as spectral-based acoustic features. MFCC, a well-established acoustic feature, has been extensively employed in training diverse machine learning models for voice identification, as demonstrated by Ali et al. 15]. Additionally, Hazra et al. [16] leveraged MFCC to train various deep learning models for the discernment of emotions in human speech. These methodologies underscore the versatility and efficacy of MFCC in diverse applications within the realm of acoustic feature-based modelling. Based on the results reported by Pyrovolakis et al. [17], which showed that combining many spectral-based acoustic features improved the accuracy of the training, we have included different spectral-based acoustic features as part of the dataset.

## 6 Methods

The dataset creation process involved distinct stages: data acquisition, data preprocessing, and data annotation, as elucidated by Gandhi et al. [18]. Each of these stages will be comprehensively addressed, incorporating the recommended fusion method tailored for multimodal music information retrieval applications utilizing the dataset.

### 6.1 Data acquisition

Like other multimodal datasets, the videos for this dataset were sourced from the social media platform, YouTube, as detailed by Zadeh et al. [11]. Various search phrases were employed on YouTube, in conjunction with savefrom.net, a video downloader site, and a Python script download.ipynb, to procure Sotho-Tswana musical video clips. These search phrases encompassed, among others, “Traditional Sotho-Tswana Music,” “Cultural Music in Sotho-Tswana Languages,” “Church Music in Sotho-Tswana Languages,” “Gospel Music in Sotho-Tswana Languages,” and “Sotho-Tswana Songs.” Native speakers proficient in the Sotho-Tswana languages were enlisted to discern and identify relevant musical videos within the dataset. A total of 150 video clips were initially downloaded in the MP4 file format through this process.

### 6.2 Data preprocessing

Musical video clips containing solely instrumentals, devoid of spoken words in Sotho-Tswana languages, were excluded, as were those in which the sound of instruments rendered it challenging to distinctly identify the language and words employed in the music composition. Similarly, musical videos with a duration of less than 15 s were discarded because the duration of such videos would be too short to discern the sentiment and genre of such videos during annotation. Additionally, musical videos exceeding a duration of two hours were omitted due to constraints associated with storage capacity and CPU processing time required for their handling.

Given the disparate durations of the downloaded musical videos, they were partitioned into fifteen-second segments, resulting in a total of 1861 segments of musical video clips. This was necessary to improve the performance during model training and evaluation, (Kiranyaz et al. [1]). This segmentation mirrors the approach employed in the creation of the CMU-MOSI multimodal video dataset (Zadeh et al. [10]).

### 6.3 Data annotation

**Fig. 3:**
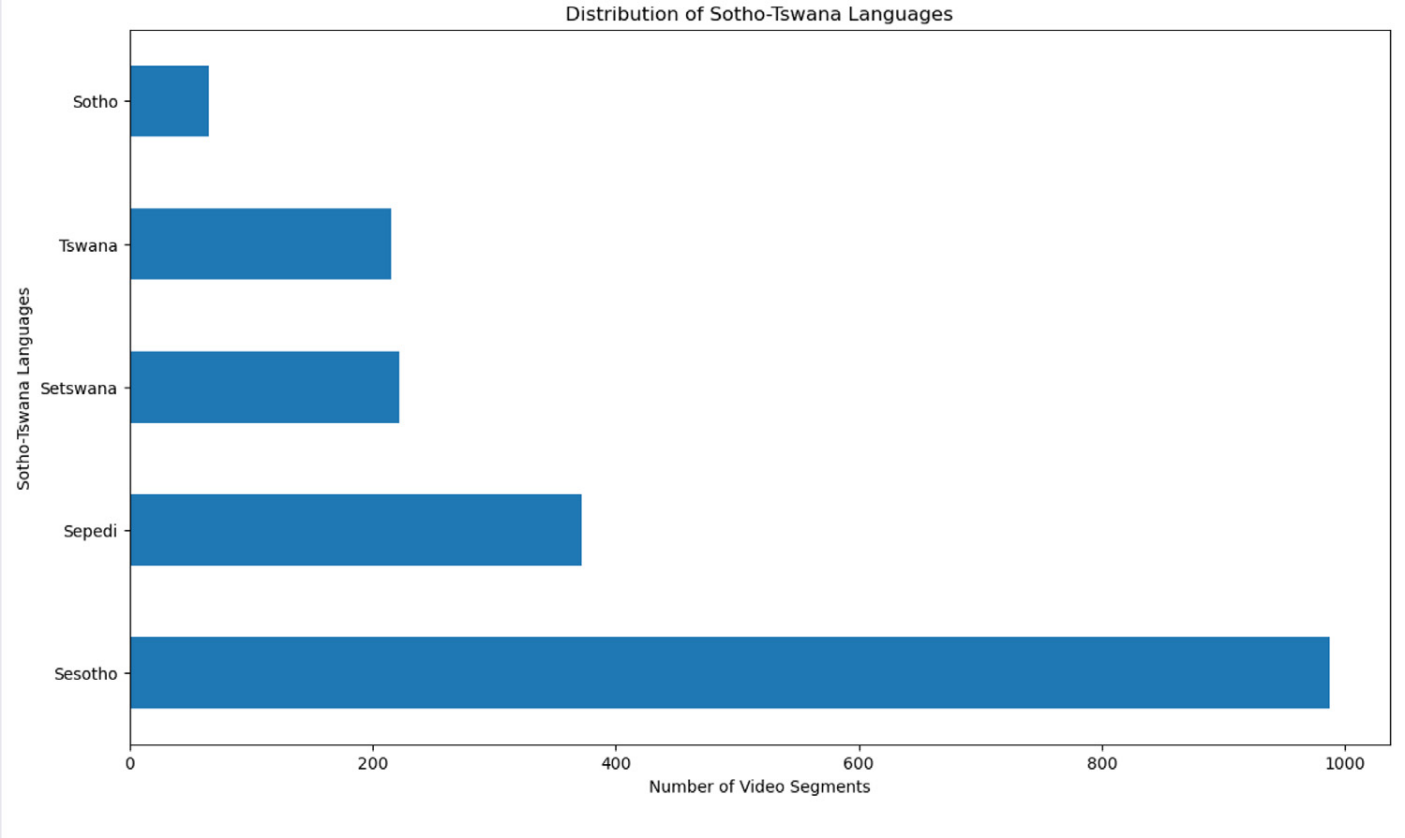
Distribution of several video segments based on Sotho-Tswana languages.

**Fig. 4:**
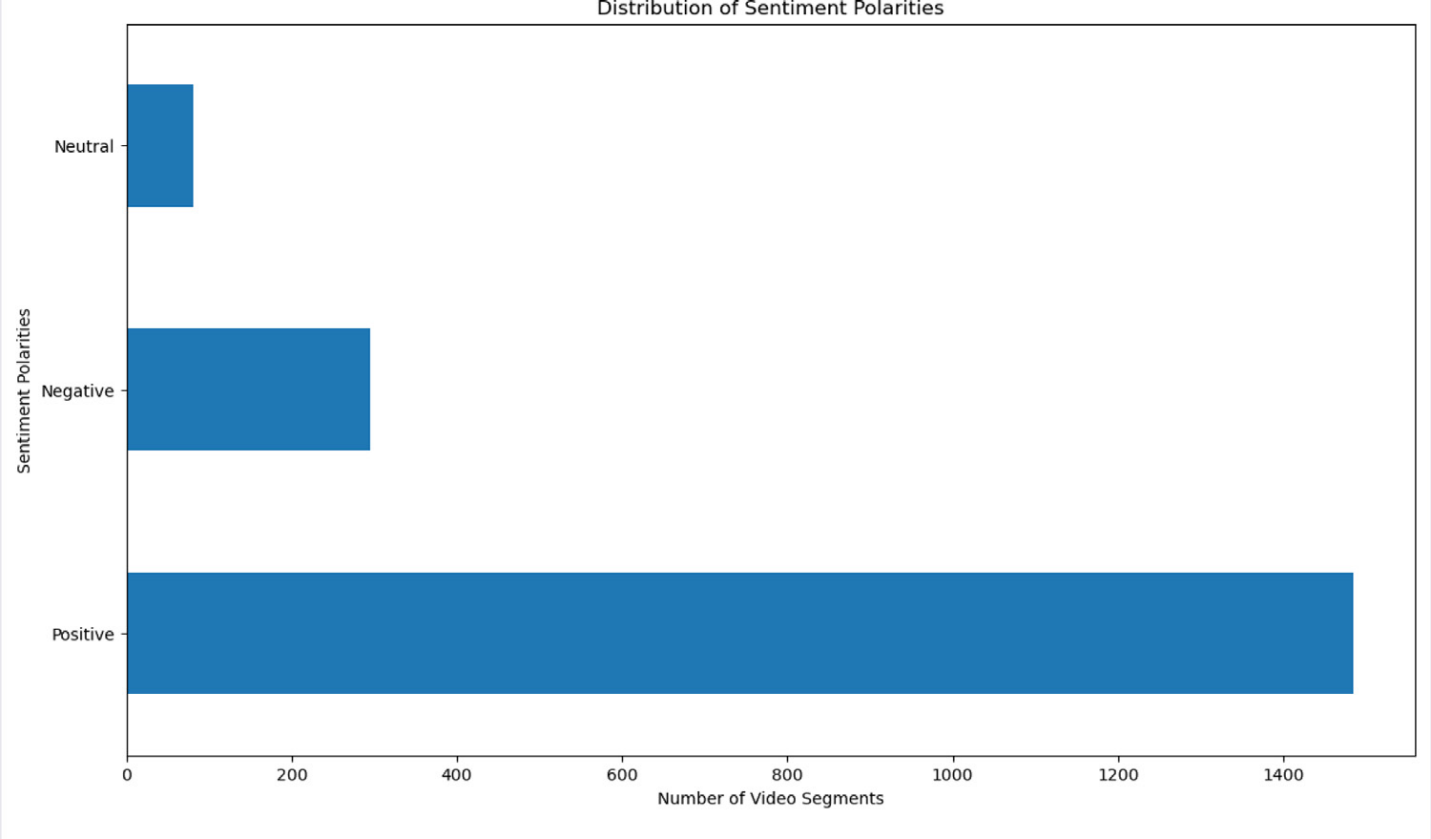
Distribution of several video segments based on sentiment polarity.

**Fig. 5:**
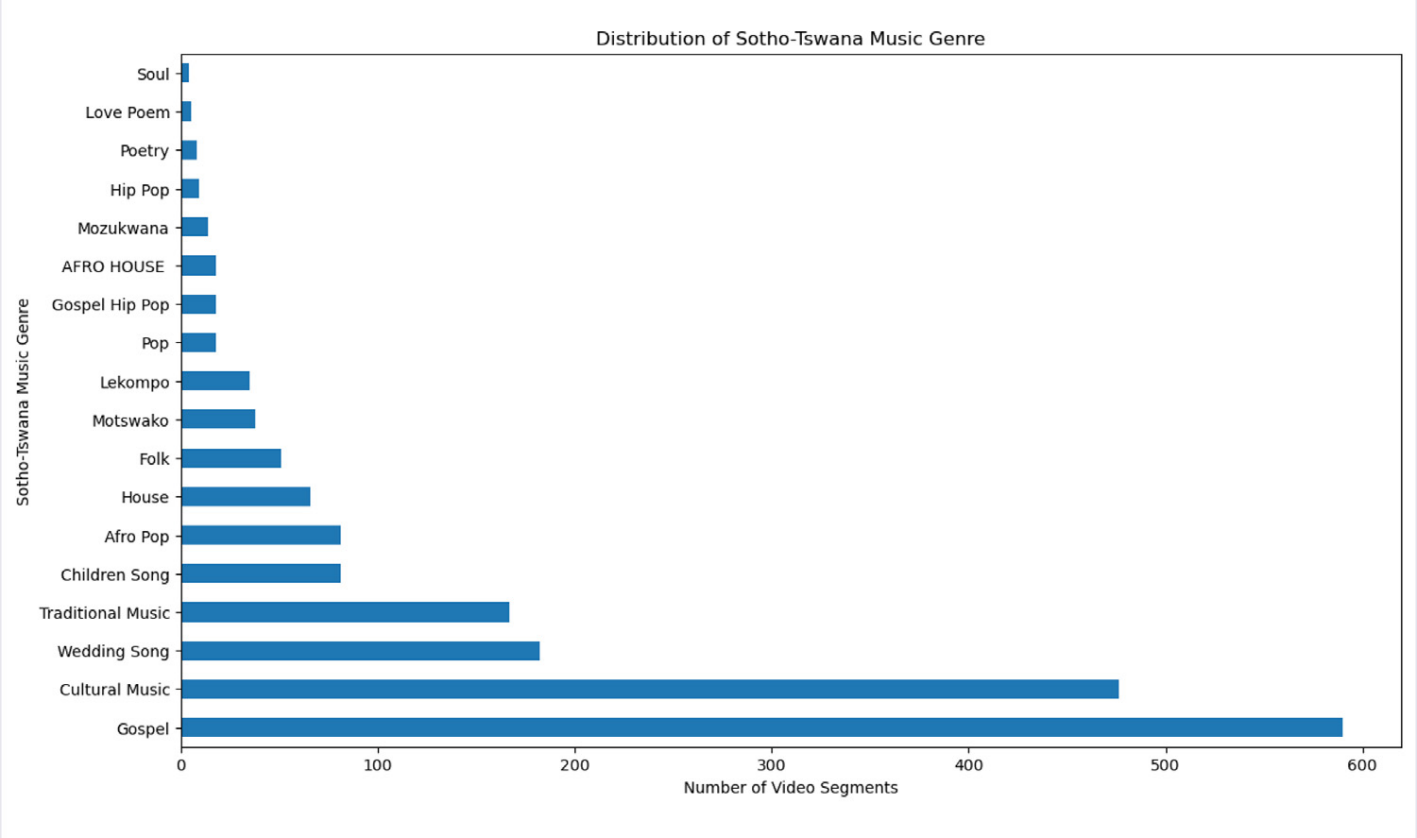
Distribution of several video segments based on the Sotho-Tswana music genre.

This stage encompasses the labelling of various metadata of the dataset. Part of the metadata that were annotated for each video included language, lyrics, genre, and sentiment. The annotations of these metadata were done manually by native speakers of Sotho-Tswana languages, who understand the musical culture and tradition of the Sotho-Tswana people, with the ability to translate lyrics of Sotho-Tswana music into English. They listened to and watched each of the downloaded musical videos from beginning to end. Afterward, they determined the language of the musical video, translated the lyrics into English, and determined sentiment polarity based on the aspect of moral and cultural values of the Sotho-Tswana people, together with the genre of the music. The annotators also explained the meaning of the lyrics of the musical video. Since each segment of the video is from a particular downloaded video, therefore, the annotations for the segments of the same downloaded video are the same. Fig. 3, Fig. 4, Fig. 5 show the distributions of the segments of the video based on language, sentiment polarity, and genre, respectively.

Multimodal datasets, as exemplified by Zadeh et al. [11] and Zadeh et al. [10], adopted a unified single annotation approach for each dataset instance. This methodology involves combining both modalities to derive a singular annotation for each instance. Two annotators listened to and watched each of the downloaded raw video clips, determined the language used, translated the lyrics of the song into English, determined the genre that it belongs to, based on the Sotho-Tswana music genres, and finally determined the sentiment polarity of the music, based on the aspect of moral and cultural values of the Sotho-Tswana people.

While our dataset, Oguike et al. [19], employed a single class annotation for these three modalities (text [lyrics], audio [voice], and visual [picture] modalities), Zadeh et al. [11] incorporated both class annotation and an estimation of the strength of the class annotation.

### 6.4 Recommended fusion method

The recommended approach for integrating features from the textual, visual, and audio modalities of this dataset is the decision-level (late) fusion method. This recommendation is grounded in its superiority over alternative fusion methods, offering advantages such as ease of training, enhanced flexibility, and simplicity, as highlighted by Pandeya et al. [20]. In alignment with this fusion method, the dataset was configured by segregating the textual and audio modalities from the visual modality. The intent is to independently train each modality and subsequently employ late fusion to amalgamate the outcomes of the training, as detailed by Gandhi et al. [18].

## Limitations

One of the limitations is the limited storage and processing capacity of the computer system used to create the dataset. With 98 downloaded musical videos of different durations, they were segmented into 1861 video clips of equal fifteen-second durations. Each of these 1861 segments of video clips is to be split into frames/images to obtain the sequence of images/frames for each video. This will result in many images/frames that will not be able to be stored or processed on a computer system with moderate storage and processing capacity, even with the use of Google Colab. Based on this limitation, we advise the user to use a random sample of segments of video clips instead of using all the 1861 segments of video clips.

## Ethics Statement

Though the videos were downloaded from YouTube, we have only provided the links to the videos in the dataset, without distributing them on the public repository.

## Declaration of Generative AI and AI-Assisted Technologies in the Writing Process

During the preparation of this work, the author(s) used ChatGPT to improve language and readability. After using ChatGPT, the author(s) reviewed and edited the content as needed and take full responsibility for the content of the publication.

## CRediT authorship contribution statement

**Osondu Oguike:** Methodology, Software, Validation, Formal analysis, Investigation, Data curation, Writing – original draft, Visualization. **Mpho Primus:** Conceptualization, Resources, Supervision, Project administration, Funding acquisition, Writing – review & editing.

## Data Availability

A Dataset for Multimodal Music Information Retrieval of Sotho-Tswana Music Videos (Original data) (Mendeley Data).
